# Searching for physical principles of morphogenesis

**DOI:** 10.1242/dev.204894

**Published:** 2025-11-13

**Authors:** Nikolas Claussen, Fridtjof Brauns, Sebastian J. Streichan

**Affiliations:** ^1^ Department of Physics, University of California Santa Barbara, Santa Barbara, CA 93106, USA; ^2^ Kavli Institute for Theoretical Physics, University of California, Santa Barbara, Santa Barbara, CA 93106, USA

**Keywords:** Morphogenesis, Physics, Theory in development, Differentiation, Mechanics

## Abstract

In morphogenesis, biology uses physics to sculpt organs. Understanding this fascinating process requires interdisciplinary collaboration. We highlight recent work in the ‘physics of development’, with a focus on the interplay between quantitative experiments and mathematical theory. We argue that the role of theory in developmental biology lies in identifying and describing dynamical mechanisms – from the processing of positional information to mechanical pattern formation – independently of their molecular implementation. This level of abstraction provides a structured approach to embrace developmental complexity, but can propose experimental tests to verify its key tenets. We conclude with a sketch of future research perspectives in emerging synthetic morphogenesis systems, which may serve as a platform to distill principles of developmental self-organization.

## Teaser

**Summary:** This Spotlight highlights recent work in the ‘physics of development’, with a focus on the interplay between quantitative experiments and mathematical theory, and presents an overview of future research perspectives in emerging synthetic morphogenesis systems.

## Introduction

Last year marked the 100th anniversary of the discovery of the Spemann–Mangold organizer (Spemann and Mangold, 1924) (the signaling center establishing the body axes of the frog *Xenopus laevis*). These experiments provided some of the first hints on how the many cells of an embryo lay down a well-proportioned body plan. Last year also marked another anniversary – 40 years since the ‘Heidelberg screen’, which laid the foundation of much of our understanding of the genetic basis of early development (Nuesslein-Volhard et al., 1984). A crucial factor in its success was the focus on single-gene, embryonic lethal mutations: an endpoint assay from which one can read off striking, unambiguous morphological phenotypes such as the deletion of all even-numbered body segments of the fruit fly larva. With knowledge of the genes determining the body plan in hand, the next step in deciphering development requires an understanding of how a small set of transcription factors orchestrates the dynamic processes that shape the body. This means coming to terms with the full complexity of development, which involves dynamic interactions between a large number of molecular players and physical factors. Making sense of this complexity will require moving beyond endpoint assays and focusing on the dynamical processes, from tissue flows to cell fate decision making, that take the embryo from egg to adult. We believe that the time is ripe to bring together the striking phenomena from classic embryological studies (such as the organizer) and our improved understanding of the underlying molecular and genetic machinery.

To create new experimental techniques and new ways of making sense of complex, dynamic phenomena, researchers in developmental biology have taken advantage of advances across scientific disciplines. During development, in particular morphogenesis, cells generate forces, sculpt geometry and establish patterns through the diffusion of morphogens. These examples highlight that development necessarily involves physics, and answering its fundamental questions will require interdisciplinary collaboration. In this article, we discuss some recent work in the field of ‘physics of development’ to highlight how quantitative biology experiments combined with physics-based theory can fruitfully address the dynamics of development.

From a practical point of view, physicists have contributed many useful tools to the arsenal of developmental biology, from new microscopy methods (Hell and Wichmann, 1994; Chen et al., 2014) to tools for image and data analysis (Blanchard et al., 2009; Etournay et al., 2016). Biology, on the other hand, can promise physicists a rich set of new physical phenomena not possible in inert materials, for example, the biomechanics of the cytoskeleton (Prost et al., 2015). But the role of physics in development goes further. It is also the scientific methods of physics, in particular its theoretical and mathematical tools, that are useful to understand development. Such theoretical approaches go back to the seminal works of Turing on pattern formation (Turing, 1952), or Oster on tissue mechanics (Oster et al., 1983), to name just two examples. The tools of modern microscopy and molecular biology bring renewed opportunities to test, refine, or falsify, such ideas. At the heart of physics is the combination of quantitative experiments, basic theoretical principles and minimal mathematical models that link these principles to experiments by making quantitative predictions.

First-principles-based biophysical modeling has shed light, for example, on the behavior of DNA polymers (Vologodskii, 2015) or molecular motors (Bustamante et al., 2001), often leveraging experiments on individual, purified molecules. However, the biological reality of most *in vivo* systems is much more complex, involving thousands of known and unknown biomolecules, the concentrations and interactions of which are difficult to measure. Therefore, the ‘fundamental constants’ of cellular biochemistry are too incompletely known for modeling to take into account all molecular details of a system.

Instead, in such situations, we believe that the task of a model is to quantitatively connect two different aspects of a biological system, for example the cell and the tissue scale of a developing embryo, or the outcomes of a cellular differentiation process and its transient dynamics. Models should be designed to cope with our incomplete knowledge of molecular details; their parameters (ideally small in number) are fitted to one experiment before they can be used to predict another. Such models are loosely called phenomenological in the physics tradition. For example, thermodynamics explains the behavior of heat, pressure and temperature without requiring an atom-level description of matter. In fact, thermodynamic laws were successfully used to design steam engines before the discovery of atoms. We believe that this type of abstraction – decoupling a conceptual, systems-level notion of what a biological mechanism does from how it is implemented molecularly – is essential to make sense of the complexity of development.

For us, theory is the combination of a set of basic principles with quantitative, mathematical models. The role of these mathematical models lies in linking ideas and hypotheses with experiments. Mathematical machinery can derive unexpected outcomes from one's assumptions, and make quantitative, rather than qualitative ‘zero/one’ (‘dead/alive’), predictions. This allows making use of experiments with subtle or variable outcomes, such as partially penetrant mutants (Corson and Siggia, 2012). By turning hypothesized mechanisms into equations, we can find new ways to test them and identify the key control parameters that determine the behavior of a system. Success with this approach requires strong feedback between experiment and theory. Experiments can be designed to be analyzed through the lens of theory. Here, theory can act analogously to ‘super-resolution’ techniques in microscopy, which deblur images by exploiting knowledge of the physical structure of fluorophores (Betzig et al., 2006). Theory can extract more insight from experimental data than expected at first sight by mathematically formulating scientific hypotheses. Theory, in turn, has to reflect new experimental findings and be able to propose experiments that verify its key tenets.

A phenomenological theory always has to be thought of together with the quantitative experiment that ‘measures something as a function of something else’ (a quote from B. Shraiman) – tissue flow as a function of motor molecule activity, for example – to determine the model parameters and test the proposed functional relationship. In the following three case studies, we aim to showcase some of the theoretical principles that this physics-motivated approach has brought to bear on development and the quantitative experiments to test them.

## Three case studies

### Positional information – optimality principles

Biological systems are selected for by evolution, leading to the hypothesis that they carry out their ‘tasks’ as well as possible. By mathematical formulation of optimality principles, one can find theoretically optimal solutions (often, by borrowing calculation techniques from physics) and use them to predict how a biological system should behave.

**Fig. 1.**
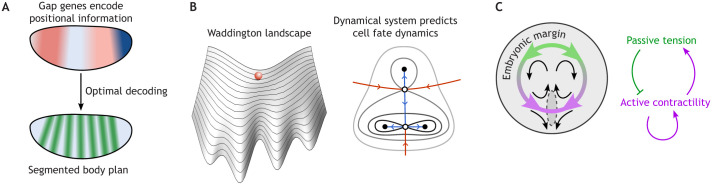
**Examples of physics-inspired theory in developmental biology.** (A) Regions in the early *Drosophila* embryo express different levels of the four gap genes, depending on their position along the anterior-posterior (AP) axis (top). Petkova et al. (2019) postulated that cells decode the gap gene concentrations to infer their AP positions using the mathematically optimal strategy. This leads to a predictive model of how the embryo's segmented body plan is altered in mutants with different gap gene patterns. The striped pattern of the downstream pair-rule genes serves as the experimental readout (bottom). Both top and bottom panels show idealized sketches not equivalent to the *in vivo* expression patterns. (B) Waddington (2014) compared differentiating cells to a marble rolling downhill, with valleys in the landscape representing distinct fates (left). Rand et al. (2021) used dynamical systems theory to compile a library of all possible dynamical motifs of three-way fate decisions, turning the Waddington landscape into predictive, mathematical models. In the example shown (right), black lines are landscape contours, stable (black dots) and unstable (white dots) fixed points correspond to terminal fates and decision points, and red and blue lines to the trajectories connecting them. (C) Amniote embryonic disk. In quail, contraction along the embryonic margin orchestrates tissue flow during gastrulation (left) (Saadaoui et al., 2020). Active contractility self-reinforces, while tissue tension represses contractility (right) (Caldarelli et al., 2024). Since mechanical tension spreads rapidly around the embryo margin, it can act as a long-range inhibitor in a mechanical Turing pattern, ensuring the formation of exactly one primitive streak.

A beautiful example of this angle of attack is the work of Petkova and colleagues on cell fate specification along the *Drosophila* embryo anterior-posterior (AP) axis (Petkova et al., 2019). At the early blastoderm stage, each cell can ‘measure’ the expression levels of the four gap genes within itself. The cell needs to make use of this noisy information to decide where along the AP axis it is located, and hence which fate it should adopt (Fig. 1A).

Petkova and colleagues posited that the cell gene regulatory networks carry out this inference task in the mathematically optimal way, resulting in a model of how gap genes determine downstream fate. To determine the model's free parameters, the authors used quantitative fluorescence microscopy of gap gene and fate markers levels in wild-type embryos. Strikingly, the calibrated model was able to accurately predict the downstream fate pattern for six different maternal mutants in which the gap gene pattern is strongly perturbed. This approach did not incorporate knowledge of molecular details!

These results shed light on what the molecular mechanisms governing AP fate specification do, even if they do not explain how. Yet knowing what is often helpful in figuring out how: in follow-up work, Sokolowski and colleagues used the same optimality principles to develop a mechanistic model of the genetic circuitry that reads out gap-gene concentrations within the cell (Sokolowski et al., 2025). However, we caution that biological systems need not be ‘optimal’ in any obvious sense; indeed, understanding what a biological system is ‘optimized’ for is as much the goal as the starting point of a theoretical investigation.

### Topology of cell differentiation – models from minimal motifs

An orthogonal approach to optimality principles (‘what is best?’) lies in the idea of classifying all potential ways a biological system could function (‘what is possible?’). Is there a set of minimal mathematical motifs from which, like Lego blocks, the biological system is assembled?

Taking this point of view, Rand and coworkers have attacked the problem of cell differentiation. This process is often conceived of in terms of the Waddington landscape, which compares a differentiating cell to a marble rolling downhill in a landscape in which valleys represent distinct fates (Waddington, 2014) (Fig. 1B). Building on work by Corson and Siggia (2012, 2017), Rand and colleagues brought to bear powerful mathematical results from the theory of dynamical systems. These results imply that the possibilities for differentiation dynamics are, in fact, highly restricted (Rand et al., 2021) and that these dynamics can indeed be faithfully represented by 2D landscapes. They compiled a list of all possible dynamical motifs of three-way fate decisions. These motifs are enumerated not through the ‘wiring’ of signaling pathways but through the geometry and topology of the dynamical landscape. For example, how are the different landscape valleys (corresponding to cell fates) connected? Rand and colleagues' motifs describe differentiation dynamics decoupled from the underlying molecular realization. A helpful analogy is the distinction between software and hardware in computer programming – the former describes what a program does, the latter its electronic implementation (Pezzulo and Levin, 2016). The same software can run on different hardware, and vice versa, the same hardware can execute different software (much like the same transcription factor can play different roles in different processes).

This work turns the Waddington landscape from a metaphor into a concrete mathematical model and, therefore, becomes directly testable by a quantitative experiment. Similar to how a chemist can combine limited information with the laws of stoichiometry to determine the nature of a chemical reaction, a small number of experiments can now be used to decide to which motif a fate decision process corresponds. The resulting model can then be used to make new predictions – for instance, on the dynamics of differentiation (what influence does the timing of a morphogen signal have on its effect?), or on the ‘epistatic’ joint effects of combining multiple morphogens or mutations (by epistasis we mean that the effect of a combined perturbation is different from the ‘sum’ of the individual effects).

The flexibility of stem cell systems – where the dosage and timing of differentiation factors can be modulated at will – makes them an ideal system to test Rand and colleagues' ideas. Sáez and colleagues demonstrated the predictivity of this approach in the context of the differentiation of mouse pluripotent stem cells to mesoderm and neural fates (Sáez et al., 2022). The ‘map’ of the differentiation landscape that such models provide may, in turn, prove useful to engineer new differentiation and organoid protocols. Crucially, this phenomenological approach can make useful predictions, even when knowledge about the underlying molecular details is scarce.

### Tissue mechanics – constraints from physical laws

The examples above take an abstract point of view of the embryo as a dynamical and information-processing system. Yet, during development, organisms must carry out the very concrete, physical task of morphogenesis: shaping tissue geometry by creating physical forces and tuning material properties via adhesion, motor and structural proteins. Morphogenesis is subject to physical constraints: cells and tissues obey the laws of mechanics, fluid dynamics and geometry. Understanding and modeling these laws is essential. They determine how action at a cellular level (such as activation of motor molecules) translates into large-scale morphogenetic results (Heisenberg and Bellaïche, 2013).

Some of our work has been along these lines: we analyzed tissue dynamics during *Drosophila* gastrulation, with the surprising conclusion that cellular packing order is required for local contractile forces to lead to large-scale convergent extension (Claussen et al., 2024). Conversely, thinking about development as a physical process has predicted new biological requirements. For example, to achieve mechanical stability, tissues need to implement negative mechanical feedback loops (Noll et al., 2017) (analogous mechanisms exist in skeletal muscles, like the stretch reflex, which modulates tension to automatically balance the muscle load). These predictions have been detected with quantitative experiments (Gustafson et al., 2022; Nishizawa et al., 2023). Together, these results indicate that morphogenesis is coordinated by a combination of negative feedback for stability and positive feedback to drive tissue flow (Claussen et al., 2024). Chan and colleagues have shown that mechanical feedback can also serve as a means to solve one of the trickiest issues of morphogenesis: size control (Shraiman, 2005). By sensing both pressure and tension within the mouse blastocyst, embryonic cells can infer and control the diameter (Chan et al., 2019). More generally, the shape of an organism must be continuously maintained, for example in the face of cell turnover, and mechano-sensation likely plays a crucial role in this process (Harris, 2018).

Recent work from Caldarelli and colleagues*.* brings all of these threads together (Caldarelli et al., 2024). Previous results have shown that localized contraction in the margin of the quail embryo drives large-scale tissue flows during gastrulation (Saadaoui et al., 2020) (Fig. 1C). But how is contractile activity coordinated across the embryo to ensure the formation of exactly one primitive streak? Caldarelli and colleagues identified a mechanical feedback loop: contraction self-activates while tissue tension inhibits contraction. Since mechanical tension spreads rapidly, it can act as a long-range inhibitor of primitive streak formation in a mechanical Turing pattern. This mechanism coordinates morphogenesis across the tissue and leads to the emergence of two correctly proportioned embryos after cutting the embryonic disk in half. Cutting stops the propagation of inhibitory mechanical tension, allowing a primitive streak to emerge in both halves. Their quantitative, physics-based model for tissue flow allowed the authors to make stringent tests of the proposed mechanism by comparing tissue flow in mechanically perturbed embryos with model predictions. Crucially, the expression of key ectodermal and mesodermal markers is modulated by tension, meaning mechanics determines the positioning of embryonic territories. Morphogenesis appears not as a top-down readout of genetic patterning, but as a process of mechanical self-organization, combining physical laws and biological dynamics (Hannezo and Heisenberg, 2019).

In addition to their biological interest, these studies have uncovered physical and mechanical phenomena of independent interest to physicists. Tissue mechanics has close links to ‘active matter’ physics, which studies systems in which building blocks perform mechanical work on the microscale. In striking contrast to morphogenesis, active matter systems often display chaotic dynamics (Sanchez et al., 2012), and taming this chaos via biology-inspired feedback loops is an area of ongoing research (Nishiyama et al., 2025).

## Outlook

These theoretical principles provide not a complete *ab initio* description of development, but rather a bridge, for example, from the behavior at the cell scale to the results at the embryo scale. Yet they allow us to make sense of highly counterintuitive phenomena, such as the ability of an embryo to reorganize its incipient body plan after being cut in two. Each of them involved close collaboration between theory and quantitative experiments. The above examples only scratch the surface of the body of excellent work by many groups across physics and biology, to which we cannot do justice in this perspective. What can the future hold for the physics of development?

In other disciplines of physics, experimental samples are specifically designed to create theoretically hypothesized phenomena. Advances in stem-cell-based organoids and embryoids may mean that the time is ripe to take a similar approach to developmental biology. What are the design principles of developmental self-organization? And can we understand them not by investigating model organisms, but by building minimal, synthetic systems, whose design is guided by theoretical hypotheses? From this point of view, the merit of an organoid system is not necessarily in how well it reproduces an *in vivo* system, but in the light it sheds on general principles of development. For example, work in gastruloids has provided insights into the self-organization of morphogen gradients (McNamara et al., 2024).

One of the most remarkable aspects of development is canalization, manifested, for example, by homeotic mutations, where altering a single gene causes an embryo's cells to converge to a different, but perfectly formed anatomical structure. Yet, this robustness is also a challenge for our understanding: through the vast space of possibilities (‘phase space’ in physics parlance) of development, a model organism traces out only a single trajectory. Synthetic systems offer the potential to systematically map out this phase space by tuning the initial conditions and mechano-chemical signals acting on developmental trajectories. The flip side of this promise is that organoids do not feature the built-in robustness of evolved organisms. Using synthetic platforms requires experimental techniques that minimize unintended sample-to-sample variability – the ability to ‘engineer’ canalization. A key step in this direction was taken by Warmflash and colleagues using micropatterned substrates to precisely control the geometry of stem cell colonies, enabling the reproducible creation of organoids that spontaneously, yet robustly, differentiate into a fate pattern resembling the early human embryo (Warmflash et al., 2014).

This protocol generates two-dimensional stem-cell colonies that do not undergo large shape changes. Remarkably, with often minimal additional input, these organoids can self-organize drastic morphogenetic reshaping, for example recapitulating the morphogenesis of the neural tube (Karzbrun et al., 2021). Via a radial gradient of the morphogen BMP, the stem cell sheet is differentiated into neural and surface ectoderm on the inside and outside, respectively. The structure subsequently folds and forms a closed tube of neural tissue, covered by surface ectoderm. The high level of control (e.g. genetic perturbations) and accessibility to mechanical measurement (e.g. traction force microscopy) afforded by organoids promise to shed light on the interplay of genetics and mechanics that underlies development. We believe that the next frontier lies in the combination of the fate and morphogenesis axes of developmental phase space in organoid platforms (Karzbrun et al., 2021; Pineau et al., 2025). Indeed, in the work of Caldarelli and colleagues reviewed above, we see how the coordinated formation of the primitive streak emerges dynamically through the interplay of mechanics and signaling. The ‘embryonic organizer’ turns out to be just as much a dynamical, mechanical process as a set of molecular and genetic factors. The organizer may be considered as an ‘emergent’ structure; i.e., a phenomenon that is more than the sum of its constituent parts (Anderson, 1972). Components (e.g. genes) of an emergent structure cannot be understood in isolation but have strong ‘epistatic’ interactions with one another.

**Fig. 2.**
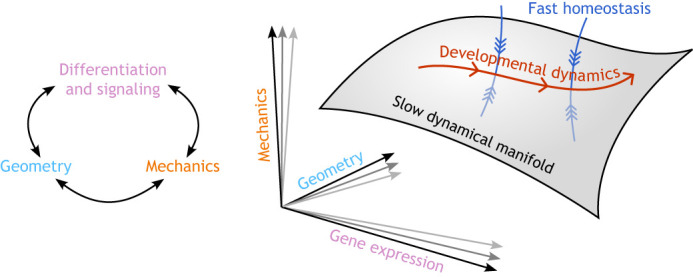
**A sketch of developmental phase space.** Development takes place in a high-dimensional ‘phase space’, defined by the gene expression profile, shape and mechanical state of embryonic cells. Additionally, cells exert tight control over their electrophysiological state (e.g. membrane potential), defining an additional ‘physical axis’. Mechanical and electrical equilibration are often the fastest timescale in developmental systems. Homeostatic feedback loops tightly coordinate these different factors, for example, by ensuring that genes for proteins that work together are co-expressed. However, instead of freezing the embryo into a single homeostatic set point, development is canalized onto a low-dimensional manifold (generalized surface), within which the slower developmental processes play out. The axes of this curved dynamical manifold are emergent, strongly correlated combinations of many different developmental factors.

Turning the notion of emergence into precise and testable models is a key challenge for theorists. A notable example is the theoretical principles that underlie developmental canalization (Russo et al., 2025). The many components of a developmental system are closely coordinated by homeostatic feedback. Yet, the system is not frozen – the embryo retains the ability to plastically develop from egg cell to adult. This suggests biological systems contain low-dimensional ‘attractor manifolds’. An attractor manifold is a generalization of a homeostatic setpoint. It is a region in state space towards which a biological system is driven by feedback loops. In contrast to a set point, however, this region comprises a whole (hyper-)surface of ‘accessible’ states between which the system can switch, allowing adaptation, development and evolution to unfold (Fig. 2). We believe that this notion is a promising framework to understand ‘emergence’ from a theoretical point of view. Although abstract, this framework makes concrete, general predictions; for example, low-dimensional attractor manifolds lead to ‘canalization’, where many distinct environmental and genetic perturbations lead to correlated phenotypic results (Russo et al., 2025).

What type of mechano-chemical dynamics can generate this delicate balance between robustness and plasticity embodied by low-dimensional attractors, and can such feedback loops explain the striking ability of stem-cell-based systems to self-organize? Interestingly, canalization-like phenomena can emerge in mathematical models for complex regulatory networks (Gutenkunst et al., 2007) and in machine learning (Belkin et al., 2019; Mao et al., 2024). These systems are highly robust to perturbations to most of their large number of parameters. Only a small number of emergent parameter combinations, analogous to Waddington's channels, strongly influence the model output and evolve as the model is trained. Are there common principles across systems as distant as artificial neural networks and developing embryos (Boukacem et al., 2024)? What role does the physical nature of the embryo – shape and mechanics – play in guiding development and evolution (Newman et al., 2006)? For example, recent work suggests that aspects of the *Drosophila* body plan evolved to buffer mechanical instabilities during development (Vellutini et al., 2025).

What kind of experiments can probe these questions? Work by Alba and colleagues provides an illustrative example (Alba et al., 2021). The authors measured the variance in the pattern of the *Drosophila* wing veins due to natural variability and environmental or genetic perturbations. The observed patterns were highly constrained, varying only along a single direction in ‘vein shape space’ while being highly robust in all other aspects. Interestingly, the authors suggested that the developmental trajectory of the wing through vein shape space aligns closely with this direction. The next step is to identify the specific biological coordination mechanisms that, in this example, ensure that all parts of the vein pattern vary together to form a single developmental trajectory. However, mechanistically understanding late development faces a crucial hurdle: the compounding complexity of development. For example, organ development does not start from the blank slate of a single egg cell but from a partially developed embryo. Genetic mutations generally affect both early and late development, making it challenging to disentangle differences in the developmental process and its initial conditions. Can similar morphogenetic canalization be observed in organoid systems, where initial and boundary conditions can be precisely controlled? Can the powerful tools available for stem cells be used to identify underlying biological feedback loops? Concretely, many current organoid systems show high variability from sample to sample. By studying this variability, we can hope to test the limits of developmental canalization and identify the physical principles that enable it.
